# Multiprofessional Neurorehabilitation After COVID-19 Infection Should Include Assessment of Visual Function

**DOI:** 10.1016/j.arrct.2022.100184

**Published:** 2022-01-31

**Authors:** Jan Johansson, Richard Levi, Maria Jakobsson, Stina Gunnarsson, Kersti Samuelsson

**Affiliations:** a Department of Clinical Neuroscience, Division of Eye and Vision, Karolinska Institute, Stockholm; b Department of Rehabilitation Medicine, Linköping University, Linköping; c Department of Health, Medicine, and Caring Sciences, Linköping University, Linköping, Sweden

**Keywords:** Covid-19, Ocular, Optometry, Rehabilitation, Vision, ABI, acquired brain injury, CISS, Convergence Insufficiency Symptom Survey, CPS, Clinical Progression Scale, NRS, numeric rating scale, VI, Vision Interview, VOR, vestibular ocular reflex, VV, visual vertigo

## Abstract

**Objective:**

To report vision-related symptoms and neuro-visual clinical signs in patients approximately 4 months after discharge from hospitalization after COVID-19 infection. To report on coexisting functional and activity limitations.

**Design:**

The study is part of an ambidirectional population-based cohort study.

**Setting:**

An outpatient setting in a hospital environment.

**Participants:**

Patients from a population-based cohort study including all patients with laboratory-confirmed COVID-19 admitted to hospital during a 3-month period in a health care region in Sweden. Among patients who, based on a standardized telephone interview, were identified as having persisting rehabilitation needs 4 months after discharge (n=185), several (n=57) reported vision-related symptoms. All 57 patients were invited to a neuro-visual examination. Six patients declined, 6 were unavailable, and 3 did not fulfil the inclusion criteria. Thus, 42 patients were included in the analysis (N=42).

**Interventions:**

Not applicable.

**Main Outcome Measures:**

Vision-related symptoms, neuro-visual function, and coexisting impairments affecting activities of daily life and participation.

**Results:**

A total of 31% of patients with rehabilitation needs after COVID-19 reported vision-related symptoms. Reading-related issues (73.8%), blurry vision (69.0%), and light sensitivity (66.7%) were the most common symptoms. Patients with reading-related issues showed a higher level of eye strain (*P*<.001). Neuro-visual deficits were found in 83.3% of the patients, mainly concerning eye teaming (23.1%-66.7%) and eye movement (28.6%-30.8%) functions. Patients with vision-related symptoms reported fatigue and 18 other coexisting symptoms to a greater extent (*P*≤.0001 to .049).

**Conclusions:**

Neuro-visual symptoms and signs should be considered when assessing rehabilitation needs after COVID-19. The association between vision-related issues and coexisting symptoms with an effect on body function and activity and/or participation underlines the need for multiprofessional rehabilitation assessment and intervention.

## Highlights

• Visual function should be considered when reviewing the rehabilitation needs of patients after COVID-19.
• The association between vision-related issues and coexisting symptoms with an effect on body function and activity and/or participation highlights the need for multiprofessional rehabilitation assessment and intervention after COVID-19.

The World Health Organization declared COVID-19 a pandemic in March 2020, and the virus has had, and likely will continue to have, a significant effect on the world's population as well as on health care and socioeconomic structures.^1^ The virus has been described as a multisystem disease affecting different organs and body functions, often with prolonged consequences on the health and daily life of individuals.^2^^,^^3^

The central and peripheral nervous system may be affected, leading to a wide variety of neurologic manifestations.^4^^,^^5^ Because of the heterogeneity of the manifestations, different possible pathogenic pathways have been suggested, for example, direct neural invasion, immune-mediated processes, endothelial dysfunction, and hypercoagulability.^6^, ^7^, ^8^ The increasing number of studies reporting on a variety of symptoms persisting after COVID-19, particularly cognitive impairments, underscore the need for multiprofessional rehabilitation.^3^, ^4^, ^5^^,^^9^, ^10^, ^11^

One commonly reported symptom that persists after COVID-19 is mental fatigue, often in combination with neurocognitive symptoms such as memory impairment, concentration difficulties, and/or an increased sensitivity to light and sound, all of which have an effect on a person's ability to manage their daily life.^12^ However, symptoms related to visual dysfunction have rarely been examined and described in this context. Issues pertaining to visual function^13^, ^14^, ^15^ and its combination with mental fatigue^16^^,^^17^ have been described in patients with acquired brain injuries (ABIs). By analogy, visual function should potentially be considered in the review of patients’ rehabilitation needs after COVID-19. In addition, visual impairments have been found to affect the ability to benefit from rehabilitation and resume daily activities after ABI.^18^, ^19^, ^20^, ^21^

Ophthalmic (eye-related) manifestations associated with COVID-19 may occur as a presenting feature of the disease or may develop weeks after recovery.^22^ The prevalence is reported to be low,^23^^,^^24^ and they most commonly involve the anterior part of the eye, for example, dry eyes and conjunctivitis.^23^^,^^25^^,^^26^ In addition, inner parts of the eye may be involved, for example, retinal disease.^8^^,^^22^ Neuro-ophthalmic symptoms include headache, ocular pain, visual impairment, and double vision,^24^ and the signs may involve optic neuritis, visual disturbances due to encephalopathy or stroke, cranial nerve palsies, and eye movement disorders.^8^

In addition to healthy eyes, different neuro-visual skills are required for functional vision and performance of daily activities. In this context, we refer to the ability to comfortably maintain clear, stable, and flexible vision in daily activities, occupational tasks, and interaction with others. Basic visual functions enabling this include visual acuity, contrast vision, binocular (eye teaming) functions, and eye movements.

This study reports on persisting neuro-visual function issues and symptoms after discharge from hospitalization because of COVID-19 infection. A second aim was to report on coexisting functional and activity limitations as sequelae of the infection.

## Methods

### Setting

This study is part of the Linköping COVID-19 Study, an ambidirectional population-based cohort study that included all patients with laboratory-confirmed COVID-19 admitted to hospital during a 3-month period in one of 21 health care regions in Sweden. All survivors at 4 months after discharge (n=460) were invited to participate in a structured telephone interview. A total of 433 patients participated (response rate 94%).^3^

As part of the Linköping COVID-19 Study, medical records were searched to classify the severity of COVID-19 at hospitalization according to the World Health Organization Clinical Progression Scale (CPS).^3^ Patients were classified as follows: CPS 4-5, hospitalized, moderate disease; CPS 6, hospitalized, severe disease, nonmechanically ventilated; and CPS 7-9, hospitalized severe disease.

### The telephone interview

The interview followed a standardized protocol comprising 37 questions, including questions on persisting symptoms from COVID-19 on body function (n=25) as well as activity and/or participation (n=12), referring to the International Classification of Function, Disability, and Health.^27^ Patients were instructed to only report new or exacerbated symptoms in relation to COVID-19. For each reported symptom, the patient was asked to estimate the effect on everyday life on a scale from 1-5 (1, no effect; 2, to a minor degree; 3, to some degree; 4, to a high degree; 5, to a very high degree). Eight questions concerned vision-related symptoms (shown in table 1) and were selected based on symptoms commonly found in individuals undergoing rehabilitation because of ABI or disease.^14^^,^^28^

**Table 1:** Responses from the telephone interview for all patients invited to a vision assessment, grouped by the severity of COVID-19 at hospitalization according to the WHO CPS

Question	Responded Yes and Graded Certain to High Effect			
	All (N=42) (%)	WHO CPS 4-5 (n=29)	WHO CPS 6 (n=4)	WHO CPS 7-9 (n=9)
Do you have trouble reading a book or a newspaper?	15 (35.7)	10	2	3
Do you experience giddiness?	16 (38.1)	13	1	2
Do you experience headache?	22 (52.4)	15	2	5
Do you experience an increased sensitivity to light, eg, outdoor light or intense indoor light?	15 (35.7)	10	1	4
Do you experience blurred or double vision?	19 (45.2)	14	3	2
Do you have trouble watching television when there is a lot of movement (eg, sport activities)?	12 (28.6)	8	1	3
Do you experience discomfort in busy environments such as traffic or when there are many people moving around you?	23 (54.8)	16	3	4
Do you experience discomfort when repeatedly altering visual focus, eg, when switching eye contact in a conversation	7 (16.7)	4		3

NOTE. CPS 4-5, hospitalized, moderate disease; CPS 6, hospitalized, severe disease, nonmechanically ventilated; CPS 7-9, hospitalized severe disease.

Abbreviation: WHO, World Health Organization.

**Fig 1:**
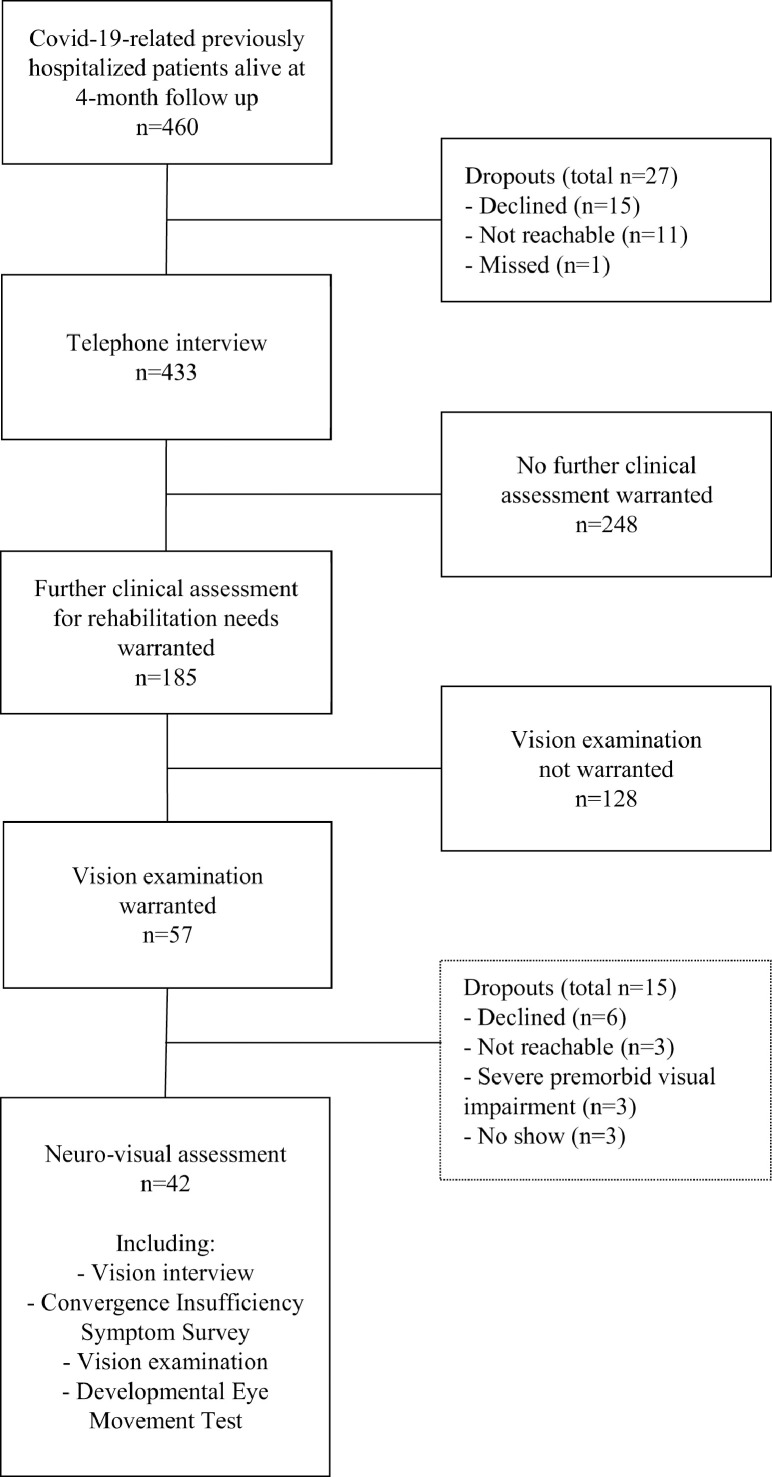
Flowchart showing the selection process of patients. A total of 657 COVID-19 related hospitalizations occurred during the study period March 1 to May 31, 2020. One hundred patients hospitalized with COVID-19 died in hospital or within 90 days of discharge. Ninety-seven patients were excluded because of comorbidities such as dementia or terminal care (n=70), underage (n=2), comorbid in-hospital death (n=16), or comorbid deaths between discharge and follow-up (n=9). The telephone interview targeted potential rehabilitation needs concerning impairments in body functions (25 items) as well as activity and participation limitations (12 items) with an effect on daily life. Eight questions in the telephone interview concerned potential vision-related issues: trouble reading, experience of giddiness, experience of headache, increased sensitivity to light, blurred or double vision, trouble watching television, discomfort in (visually) busy environments, and discomfort when altering focus. Patients who rated a significant effect on daily life for any of these symptoms were offered a neuro-visual assessment. For further details, refer to the previous publication.^3^

About 40% of all interviewed patients were identified by the interviewers and confirmed with a rehabilitation team as having a need for further clinical assessments and potentially rehabilitation interventions, based primarily on to what degree the symptoms affected daily life. Those participants were then invited to a clinical examination by a multiprofessional rehabilitation team about 5 months after discharge from hospital (fig 1). Patients who reported neuro-visual symptoms with a significant effect on daily life (3-5 on the rating scale) at the time of the interview or at the clinical visit were offered a special neuro-visual assessment approximately 5-6 months after hospitalization. Non-Swedish-speaking patients were assisted by an interpreter both at the telephone interview and the neuro-visual examination.

### Neuro-visual examination

The neuro-visual examination included a detailed self-reported assessment of vision-related symptoms, an in-depth vision examination, and an assessment of reading-related oculomotor skills.

Vision-related self-reported symptoms were assessed with the Vision Interview (VI)^28^ and the Convergence Insufficiency Symptom Survey (CISS).^29^ These documents were filled in by the patient before the vision examination. The VI is an 18-item structured questionnaire (questions shown in table 2) intended as support when reviewing visual impairments after ABI, including infection diagnoses. CISS consists of 15 questions concerning symptoms associated with reading and near activity graded on a Likert scale (0, never; 1, infrequently; 2, sometimes; 3, fairly often; 4, always). A total score of 21 or more is considered a high level of symptoms.^30^ Symptoms concerning light sensitivity and dizziness were assessed as part of the anamnesis using a selection of questions adapted from questionnaires in previous research.^31^^,^^32^

**Table 2:** Vision Interview

No.	Item	No. of Responses	Response		
			Yes	No	Don't Know
1	Have you noticed any type of vision change?	42	32	6	4
2	Do you experience double vision?	42	7	34	1
3	Do you experience reading difficulties?	42	31	11	0
4	Do you experience difficulty when moving among people and objects?	42	4	32	6
5	Do you frequently bump into people or objects?	42	11	28	3
6	Do you experience difficulty with depth perception, eg, on stairs?	42	11	28	3
7	Do you experience difficulty with eye-hand coordination, eg, when reaching for a glass?	42	4	38	0
8	Do you experience difficulty recognizing faces?	41	1	40	0
9	Do you perceive familiar faces differently?	42	6	33	3
10	Do you become more dazzled by light?	42	28	10	4
11	Do you need more light in general to see well?	38	15	17	6
12	Do you need more light while reading?	40	23	12	5
13	Is your vision blurrier now?	42	29	9	4
14	Is your color perception different now?	41	3	30	8
15	Have you experienced any visual phenomenon?	39	28	11	0
16	Have you experienced any other visual concern?	39	10	24	5
17	Is your visual field affected?	39	5	27	7
18	Have you had an eye or vision examination since the injury?	39	9	30	0

The in-depth vision examination was performed by a licensed optometrist with neuro-rehabilitation experience. It included a brief anamnesis, visual acuity at near and far, eye motility, stereovision, eye teaming function, eye movement function, accommodation, and contrast vision (table 3).^33^ Eye motility and eye movement function were clinically examined using criteria adapted from the Ocular Motor Score.^34^ Exclusion criteria were premorbid eye disease and neurologic disorder causing severe visual impairment.

**Table 3:** List of visual functions examined, assessment methods, and assessment criteria

Visual function	Method	Criteria
Visual acuity	Snellen Vison Chart at 3 m	Monocular decimal acuity 1.0 or better
Near visual acuity	Near vision chart at 40 cm	Binocular acuity of 5 p or better with or without reading aids
Eye motility	Clinical assessment (OMS)	(A) No restrictions in 8 gaze directions
		(B) Some form of motility restriction, over or under functions
		(C) Advanced motility restriction/apraxia
Stereovision	Lang II stereotest	Positive (identifies all symbols), negative (sees only star), partially positive (cannot identify the symbols)
Binocular function		
Near point of convergence	RAF near point ruler	Near point of convergence >10 cm
Convergence facility	3 base in 12 base out prism	Age 39 y or younger, minimum 11 cycles/min
		Age 40 y or older min 7 cycles/min
Fusion vergences at distance, 3 m		Fusion vergence width <19 prism diopters
Fusion vergences at near, 40 cm		Fusion vergence width <27 prism diopters
Eye movements		
Fixation	Observation	(A) Stabile fixation in primary and 6 gaze directions
		(B) Saccadic intrusions, detectable drifts
		(C) Nystagmus observed
Pursuit movement	Observation	(A) No intruding saccades, no head movements
		(B) 3-4 intruding saccades, head movement
		(C) >5 intruding saccades, large head movement
Visually induced saccades	Observation	(A) Normal latency, no obvious hypo- or hypermetria
		(B) Long latency, dysmetria, head movement
		(C) Advanced dysmetria, long latency, poor conjugacy, head movement
Self-paced saccades	Observation	(A) Normal intersaccadic latency, no obvious hypo- or hypermetria
		(B) Variable intersaccadic latency, dysmetria, head movements
		(C) Markedly prolonged latency, stops, closes eyes
Self-induced oscillating head movement, horizontal/vertical	Observation	(A) Maintains fixation, steady pace, no discomfort
		(B) Loses fixation, irregular pace, discomfort
Hand motion in visual field	Observation	(A) Maintains fixation, no discomfort
		(B) Fixation loss, discomfort, closes eyes
Accommodation	RAF near point ruler	Minimum expected amplitude according to the Hofstetter formula
Contrast Vision	Pelli-Robson charts, monocular and binocular illumination 99.9 cd/m^2^	According to normative values^33^

Abbreviations: OMS, ocular motor score; RAF, Royal Air Force.

The Developmental Eye Movement Test^35^ was used to examine oculomotor skills in a simulated reading task. The patient reads out numbers arranged vertically and horizontally on 3 different test cards. The ratio between vertical and horizontal reading duration is then calculated.

### Statistical analysis

The statistical analyses were done using SPSS Statistics 25^a^ and/or Microsoft Excel 2010.^b^ Chi-square or Fisher exact test was used for analysis of cross-tabulations of frequencies. Welch's *t* test or analysis of variance was used for comparing means. Normality tests were performed with the Kolmogorov-Smirnov method. A *P* value <.05 was set as the level of significance.

### Ethics

The study adhered to the tenets of the Declaration of Helsinki and was approved by the Swedish Ethical Review Authority (Dnr 2020-03029 and 2020-04443). All patients gave informed consent before participation.

## Results

Fifty-seven of the 185 patients invited to a clinical examination (31%) were also invited to a neuro-visual examination. A total of 42 patients attended the examination and were thus included in the analysis (table 4).

**Table 4:** Demographics

	All Patients (N=42)	WHO CPS 4-5 (n=29)	WHO CPS 6 (n=4)	WHO CPS 7-9 (n=9)
Female/male, n (%)	23/19 (54.8/45.2)	18/11 (62.1/37.9)	2/2 (50.0/50.0)	3/6 (33.3/66.7)
Age at vision examination (y), mean ±SD	53.4±13.3	51.2±14.8	60.9±3.1	57.0±8.6
Time since discharge (d), median (min-max)	162 (114-345)	178 (114-345)	157 (132-166)	154 (132-308)
Days spent in hospital, median (min-max)	5.0 (1-38)	2.0 (1-20)	15 (12-23)	19 (15-38)
Days in intensive care, median (min-max)	12 (8-31) (n=9)	- (n=0)	10 (n=1)	12.5 (8-31) (n=8)

Abbreviations: CPS, Clinical Progression Scale; WHO, World Health Organization.

The validity of the selection process of patients invited to a neuro-visual assessment was reviewed retrospectively; the responses from those invited (n=57) were compared with those not invited (n=128) for 6 of the 8 telephone interview questions about visual symptoms (questions about headache and giddiness excluded). A chi-square test showed a significant association between group and symptoms, confirming that patients invited to the vision examination were much more likely to experience visual symptoms affecting their daily life (*P*<.01).

### Telephone interview responses by patients invited to the neuro-visual examination

The number of patients who responded affirmatively to questions pertaining to visual dysfunction and rated the effect 3-5 (certain to high effect on daily life) are shown in table 1. A chi-square test showed no association between symptoms and CPS classification.

### Results from the neuro-visual examination

#### Visual symptoms according to the VI

The most reported symptoms were reading difficulties, blurry vision, dazzled by light, and an increased need for light while reading (see table 2). Patients who had experienced a visual phenomenon described it as foggy vision (n=15) or in the form of flares, dots of light, or stars in their field of vision (n=13).

#### Visual symptoms when reading and for near work according to CISS

The mean CISS score was 25.9±10.6 for the total group, suggesting an increased level of symptoms (cut off score ≥21). There was no difference between the groups based on CPS classification. For patients experiencing reading-related difficulties according to the VI (n=31), the mean CISS score was 29.7±8.8 compared with 15.2±7.3 for patients without reading-related difficulties (*P*<.01).

#### Headache

In the anamnesis, 20 patients reported headaches of greater intensity or to occur more frequently after the infection. Six patients reported the headache to be specifically associated with reading and near activity according to CISS.

#### Light sensitivity

Twenty-three patients (54.8%) experienced moderate to severe discomfort when exposed to light and that vision was degraded when exposed to light. Common factors perceived to worsen the symptoms were fatigue, time (worsens as the day goes on), watching television, and the use of a computer, tablet, or mobile phone. All types of light, indoors as well as outdoors, could be bothersome. No association between symptoms and the CPS classification was found. There was no association between light sensitivity and headache.

#### Dizziness and fatigue

Twenty-eight patients (66.7%) graded symptoms of dizziness as always present. Four patients reported that symptoms were associated with head movement, and 10 patients reported motion in the environment as the cause of symptoms. The remaining 14 patients reported that symptoms were associated with both conditions. Table 5 describes the symptoms at group level. A chi-square test of independence showed no association between symptoms and CPS classification.

**Table 5:** Dizziness associated with head movement or motion in the environment

Symptom	Always	Sometimes	Never	Avoids
Do you become dizzy				
When bending over?	5	21	16	
When rolling over?	11	16	15	
When turning your head quickly?	3	21	17	1
If you turn your head while walking?	9	16	17	
Do you have symptoms of discomfort, unsteadiness, dizziness or imbalance				
When shopping in a supermarket?	15	11	15	1
When walking along a tree-lined street?	6	4	32	
When looking at striped or very busy patterns?	8	6	28	
When watching a movie on television or scrolling on the computer screen or tablet?	15	11	15	1
When people are moving around you?	11	8	22	1
When riding as a front seat passenger?	8	8	24	1

NOTE. Eighteen patients responded that they always experienced at least 1 of the symptoms in association with head movement, eg, when changing posture or turning the head. Twenty-four patients responded that they always experienced at least 1 of the symptoms associated with motion in the environment.

Before the neuro-visual examination, each patient was asked to grade their present level of fatigue using a numeric rating scale (NRS), where 0=no fatigue and 10=extreme fatigue. This was performed to get an estimate of self-perceived fatigue on the examination day. Thirty-four patients provided a rating, resulting in a mean score of 4.8±2.8.

#### In-depth vision examination

Thirty-five patients (83.3%) had 1 or more clinical signs concerning eye teaming function, accommodative function, eye movements, or reaction to motion in the peripheral field (table 6). No associations between symptoms and CPS classification were found. The types of clinical signs are specified in table 7.

**Table 6:** Number of clinical signs found in the vision examination for all included patients and patients grouped by the severity of COVID-19 at hospitalization according to the WHO CPS

No. of Clinical Signs	All Patients (N=42)	WHO CPS 4-5 (n=29)	WHO CPS 6 (n=4)	WHO CPS 7-9 (n=9)
None	7	4	1	2
1	7	4	1	2
2	10	7	1	2
3	4	3		1
4	6	5		1
5	3	3		
6	2	1		1
7	1		1	
8	1	1		
9	1	1		

NOTE. Clinical signs are deviations in visual function, ie, where the criteria for normal function are not met. The criteria are listed in table 3, and the specific types of clinical signs found in vision examination are presented in table 7. Up to 8 clinical signs were found in a patient. CPS 4-5, hospitalized, moderate disease; CPS 6, hospitalized, severe disease, nonmechanically ventilated; CPS 7-9, hospitalized severe disease.

Abbreviation: WHO, World Health Organization**.**

**Table 7:** Clinical signs found at the vision examination in all included patients

Clinical Sign	Patients, n (%)	Comment
Refractive error (n=42)		
Suboptimal refractive correction	12 (28.6)	
Contrast sensitivity (n=26)		
Sensitivity below expected according to age	4 (15.4)	Cataracts (n=2)
Eye motility and versional (gaze) eye movements (n=42)		
Eye motility restriction	1 (2.4)	Premorbid condition
Fixation (saccadic intrusions, detectable drifts, or nystagmus)	2 (4.8)	
Smooth pursuit (intruding saccades or head movements)	4 (9.5)	
Visually induced saccades (long latency, dysmetria, head movements)	3 (7.1)	
Voluntary saccade (variable or prolonged latency, dysmetria, head movements, stops due to discomfort)	12 (28.6)	
Head movement and motion sensitivity		
VOR head movement (patient-reported discomfort or observed fixation loss) (n=39)	12 (30.8)	
Hand motion in peripheral visual field (patient-reported discomfort, observed fixation loss) (n=41)	6 (14.6)	
Binocular (eye teaming) functions (patients with stereovision, n=39)		
Reduced fusion vergences at distance (3 m)	20 (51.3)	
Reduced fusion vergences at near (40 cm)	15 (38.5)	
Reduced near point of convergence	9 (23.1)	
Reduced convergence facility	26 (66.7)	
Accommodative function (age <40 y, n=6)		
Reduced accommodative amplitude	3 (50.0)	

NOTE. Clinical signs are deviations in visual function, ie, where the criteria for normal function are not met. The criteria are listed in table 3. Eye motility concerns the ability to move the eyes in all directions without restriction or experiencing double vision. Versional, or gaze, eye movements are when the eyes move in unison in the same direction. The movements are stabilizing (fixation), rapid (saccades), or following (pursuit) and are essential for the acquisition of visual information, eg, through focusing, visual search and/or scanning of the environment and tracking of moving objects. Binocular functions concern the teaming of the eyes. Eye teaming is essential for maintaining clear and single (not double) vision and to adjust for different viewing distances. Eye teaming happens through vergence eye movements where the eyes move in opposite directions.

#### Developmental Eye Movement Test

All patients performed the Developmental Eye Movement Test. Two patients were outliers (exceeded third quartile+1.5 × IQR) and thus were excluded. There was no difference in the parameters or the ratio between patients who experienced reading issues according to the VI and patients who did not. There was no difference in the results between patients who had increased symptoms according to CISS (>21) and those who did not. A 1-way analysis of variance showed no difference in the ratio based on the CPS classification.

#### Symptoms vs clinical findings

A chi-square test for independence was performed to explore associations between symptoms according to the VI and clinical signs found in the vision examination. For reading difficulties (VI question 3), there was an association with a deficit in vestibular ocular reflex (VOR) head movement (Fisher exact test, *P*=.02). For other visual concern (VI question 16), there was an association with eye movement deficits (Fisher exact test, *P*=.04) and VOR head movement (Fisher exact test, *P*<.01). Other vision concern included blurry or foggy vision, fluttery vision, a delay in visual perception, sore eyes, watching television feels different, and impaired depth perception.

Twenty-eight patients reported always experiencing at least 1 symptom related to dizziness because of head movement, motion in the environment, or a combination. In patients who had symptoms of dizziness associated with head movement, a chi-square test for independence showed associations with deficit in VOR head movement (*P*=.01) and deficit in eye teaming width at distance (*P*=.02). No associations were found between clinical signs and dizziness associated with motion in the environment.

Eighteen patients showed 3 or more clinical signs in the vision examination. The total number of signs in this subgroup corresponded to 76.1% of the total number of signs found. When analyzing this subgroup vs the remaining group who did the vision examination there were no significant differences in sex, number of days spent in the hospital, time since discharge from the hospital, CPS classification, or need for intensive care. However, there were differences in age (mean, 48.6±14.5 years vs 56.9±11.3 years) and fatigue according to NRS (6±2.9 vs 4.1±2.5), where the subgroup was younger (*P*=.04) and had higher NRS scores (*P*=.04).

### Coexisting persisting symptoms at follow-up

The results are based on telephone interview data from patients who had a clinical team examination (n=128) vs those who also had a vision examination (n=42). The results demonstrate a difference in the distribution of symptom prevalence between the groups. Patients invited to the vision examination did report symptoms affecting their daily life to a greater extent (table 8).

**Table 8:** An analysis of the difference in rated impairments affecting daily life on body functions and activity and/or participation issues in those who were invited to a clinical team assessment (n=128) vs those who attended vision examination (n=42)

Item	n	χ^2^ Value	*P* Value
Difficulty concentrating	126/42	29.12	<.0001
Headache	126/42	19.87	<.0001
Loud sound sensitivity (phonophobia)	125/42	19.62	<.0001
Difficulty remembering	126/42	14.64	.0001
Difficulty understanding speech	123/41	14.06	.0002
Stress sensitivity/irritability	124/39	13.84	.0002
Mental slowness	126/42	13.71	.0002
Mental fatigue/fatigability	127/42	10.46	.0012
Feeling anxious	125/42	9.31	.0023
Sleep less/disturbed sleep (>2h change)	123/40	6.93	.0085
Feeling low/depressed	125/41	5.71	.0169
Weak/hoarse voice (dysphonia)	119/38	3.87	.0491
Muscular soreness/aches/cramps/discomfort	127/42	2.96	NS
Slurred/indistinct speech (dysarthria)	119/38	2.26	NS
Weakness/fatigability in arms and/or legs	128/41	1.76	NS
Sleep less/ disturbed sleep (≥2h change)	125/42	1.69	NS
Difficulty hearing	125/41	0.47	NS
Altered bodily sensation	126/42	0.26	NS
Altered smell and/or taste	94/30	0.06	NS
Difficulty swallowing	112/37	0.03	NS
Difficulty participating in social activities (socializing with friends and family)*	122/37	36.89	<.0001
Difficulty multitasking*	126/42	29.11	<.0001
Difficulty managing work/studies*	57/24	25.63	<.0001
Difficulty word-finding when speaking*	125/41	14.73	.0001
Difficulty driving a car/using public transportation*	111/38	6.47	.0110
Difficulty walking >1 km*	125/38	4.89	.0270
Difficulty being physically active*	127/42	4.7	.0302
Difficulty managing ADL*	124/39	2.49	NS
Experienced falls after discharge*	127/41	0.25	NS

Abbreviations: ADL, activities of daily living; NS, not significant.

⁎ Activity and/or participation.

## Discussion

Of the 433 patients interviewed 4 months after discharge from hospital, 13.9% were identified as experiencing a significant effect from at least 1 of the symptoms considered to indicate a neuro-visual dysfunction.

The symptom assessment indicated considerable percentages of remaining symptoms concerning reading, clarity of vision, headache, light and motion hypersensitivity, and dizziness affecting daily life. Difficulties with reading were frequent and associated with increased levels of eye strain. The analysis did show an association with deficits in VOR head movement, an important function for stabilizing gaze during acquisition of detailed visual information. The absence of further significant associations with clinical signs illustrates the challenges of linking a single function (visual function) to the complex process of reading.^36^

Blurred or doubled vision was another frequently reported symptom affecting daily life activities. There was at least 1 clinical sign in 84% of the patients, most commonly affecting eye teaming functions. The clinical signs were similar to findings in studies on patients with ABI,^13^, ^14^, ^15^ and further research is required to verify and understand the mechanisms.

A considerable number of the patients had suboptimal refractive correction for distance or near vision. Refractive correction is relevant not only for clear comfortable vision but also to optimize eye teaming functions. With the increased number of symptoms and signs found here and elsewhere,^5^^,^^24^^,^^37^ it appears that the need for proper refractive correction and/or reading aids should be considered for these patients.

Headache was reported by 47%. In patients who reported that headaches were associated with reading or near activity, obvious neuro-visual deficits and/or suboptimal correction were found. Thus, it appears that neuro-visual deficits may be part of the mechanism but given the overall constellation of symptoms, the mechanism is likely to be multifactorial.

Twenty-three patients (55%) reported discomfort in the eye or head when exposed to light, indicating that they had light sensitivity (photophobia).^38^ Common ocular conditions that may cause photophobia are dry eye or eye disease. Dry eye disease has been reported after COVID-19-infection.^25^^,^^26^ Four patients with symptoms of gritty and sore eyes, intermittent blurry vision, and light sensitivity that worsened when watching television or using a computer or mobile phone were referred for ophthalmologic assessment. Otherwise, there were no suspected signs of ocular disease in the vision examination or ocular history.

Migraine and other primary headaches are common neurologic disorders that may cause light sensitivity.^38^^,^^39^ The current analysis did not show any significant associations between headache and light sensitivity. However, high percentages of headache and light sensitivity have been reported in the acute phase^40^ and as part of the long-term symptoms^23^^,^^24^^,^^41^; therefore, further study may be warranted to explore the association.

About two-thirds of the patients reported symptoms of dizziness. Dizziness is a neurologic manifestation associated with COVID-19 with a prevalence of 0.6%-21%.^6^^,^^42^ Reports describing the mechanism are scarce because of limited research involving complete vestibular evaluations.^43^ A considerable percentage of the patients reported symptoms provoked by visual motion, suggesting visual vertigo (VV). VV is defined as dizziness provoked by visual environments with large-size repetitive or moving visual patterns.^44^ Patients typically experience dizziness and discomfort in situations such as busy crowded places, in traffic, or when walking down supermarket aisles. VV may be present in patients with a history of a peripheral vestibular disorder and especially in those with high visual dependence.^44^

A total of 83% of the patients examined had clinical signs verified with objective assessment methods. This suggests that a fairly brief battery of questions can identify patients with a potential need for intervention.

The association between fatigue and visual functions may be a subject for further study. However, the difference in the frequency of reporting cognitive impairment affecting daily life in patients with visual impairment compared with those who did not is notable. In the present study, a significant difference in self-estimated fatigue was found between patients with 3 or more clinical signs and those with 2 or less. Similar findings have been presented for patients with ABI where an association between fatigue according to the Mental Fatigue Scale and visual symptoms has been shown.^16^

Our findings suggest that visual symptoms after COVID-19 may be associated with objective visual function issues. In a clinical setting, some of these issues could be verified through visual screening or by referring to an optometrist or other eye care specialist. Important basic interventions intended to support return to daily activities should be given in an individually adapted mix of, for example, vision therapy, proper spectacle correction, customized spectacles for reading and near activity, tinted lenses, and guidance on visual ergonomics.

### Study strengths

The study was a cohort study and included all patients who were hospitalized because of COVID-19 infection in a health care region. All patients were interviewed according to a standardized protocol based on the International Classification of Function, Disability, and Health and included vision-related items. The study also included those who spoke a foreign language (via an interpreter).

### Study limitations

The final sample size (N=42) is fairly small, and further research is required to verify the findings. The interview items regarding visual symptoms were chosen based on available knowledge at the time, and some symptoms may therefore not have been captured. The selection of the study group was based on self-reported symptoms. For patients with other and more troublesome symptoms, vision-related symptoms might have been neglected and thus not reported. Patients invited for a neuro-visual assessment were younger and reported a higher level of mental fatigue than those who were not invited. Younger patients might be more affected by visual dysfunctions because of a higher expected level of activity than older patients. The vision examination did not include ophthalmologic examinations, and therefore the study does not fully take ocular health into consideration. In those patients who declined participation, it is unclear if this was because of spontaneous recovery or other reasons. Neuroimaging data were not included in this study, and therefore the study does not take neurologic diagnoses into consideration. Finally, the study included only those who rated a certain level of effect or higher, and therefore underreporting is possible.

## Conclusions

We conclude that a considerable proportion of patients (31%) with potential rehabilitation needs approximately 4 months after COVID-19 also reported vision-related symptoms. Reading-related issues, eye strain, blurry vision, and light sensitivity were the most common symptoms affecting patients’ daily life. The neuro-visual examination showed clinical signs in 83% of the patients, mainly concerning eye teaming and eye movement functions necessary to comfortably maintain clear, stable, and flexible vision. In addition, patients with vision-related symptoms reported coexisting persisting symptoms such as mental fatigue and other cognitive impairments to a greater extent than patients without vision-related symptoms, which underlines the need for future multiprofessional rehabilitation assessment and intervention.

## Suppliers

a. SPSS Statistics 25; IBM, Armonk, NY.
b. Microsoft Excel 2010; Microsoft, Seattle, WA.
